# Single-dose versus multiple-dose antibiotic prophylaxis for the surgical treatment of closed fractures: A cost-effectiveness analysis

**DOI:** 10.3109/17453671003587119

**Published:** 2010-04-06

**Authors:** Gerard P Slobogean, Peter J O'Brien, Carmen A Brauer

**Affiliations:** ^1^Department of Orthopaedics, University of British Columbia, Vancouver, BC; ^2^Department of Pediatric Orthopaedics, Alberta Children's Hospital, Calgary, AlbertaCanada

## Abstract

**Background and purpose** Recent meta-analyses have suggested similar wound infection rates when using single- or multiple-dose antibiotic prophylaxis in the operative management of closed long bone fractures. In order to assist clinicians in choosing the optimal prophylaxis strategy, we performed a cost-effectiveness analysis comparing single- and multiple-dose prophylaxis.

**Methods** A cost-effectiveness analysis comparing the two prophylactic strategies was performed using time horizons of 60 days and 1 year. Infection probabilities, costs, and quality-adjusted life days (QALD) for each strategy were estimated from the literature. All costs were reported in 2007 US dollars. A base case analysis was performed for the surgical treatment of a closed ankle fracture. Sensitivity analysis was performed for all variables, including probabilistic sensitivity analysis using Monte Carlo simulation.

**Results** Single-dose prophylaxis results in lower cost and a similar amount of quality-adjusted life days gained. The single-dose strategy had an average cost of $2,576 for an average gain of 272 QALD. Multiple doses had an average cost of $2,596 for 272 QALD gained. These results are sensitive to the incidence of surgical site infection and deep wound infection for the single-dose treatment arm. Probabilistic sensitivity analysis using all model variables also demonstrated preference for the single-dose strategy.

**Interpretation** Assuming similar infection rates between the prophylactic groups, our results suggest that single-dose prophylaxis is slightly more cost-effective than multiple-dose regimens for the treatment of closed fractures. Extensive sensitivity analysis demonstrates these results to be stable using published meta-analysis infection rates.

## Introduction

The use of prophylactic antibiotics in the surgical treatment of closed long bone fractures is well-established (Boyd et al. 1973, Burnett et al. 1980, Gatell et al. 1984); however, the duration and dosage of prophylaxis varies substantially among surgeons (Gatell et al. 1987, Buckley et al. 1990, Garotta et al. 1991). Previous meta-analyses comparing single- and multiple-dose prophylaxis for surgical fixation of fractures have failed to demonstrate the superiority of either prophylactic strategy (Southwell-Keely et al. 2004, Gillespie et al. 2006, Slobogean et al. 2008). Although a definitive prophylactic recommendation cannot be made from these studies, the pooled results demonstrate that surgical site infections in this population are uncommon and that any potential differences in infection rates between the strategies is likely to be small. Based on the low incidence of infection observed in the pooled studies, it has been suggested that over 25,000 patients would be needed to demonstrate superiority of either strategy, making a clinical trial unlikely (Slobogean et al. 2008).

Decision analysis techniques can offer an alternative method for answering clinical questions when performance of a clinical trial is not feasible (Brauer and Waters 2007). Cost-effectiveness analysis uses economic and preference-weighted health state data to mathematically model clinical decisions (Russell et al. 1996). From this analysis, a preferred treatment strategy can be suggested using commonly accepted criteria (Siegel et al. 1996, Weinstein et al. 1996). Additionally, this type of economic evaluation allows one to identify the numeric boundaries of key variables where its conclusions become unstable. A review of the basic principles and importance of economic evaluations was recently published in this journal (Dijksman et al. 2008).

In order to explore potentially small differences in efficacy between prophylactic dosing practices and to estimate the economic and quality of life implications of perioperative prophylactic decisions, we performed a cost-effectiveness analysis comparing single-dose and multiple-dose antibiotic prophylaxis for the surgical treatment of closed fractures.

## Materials and methods

### Overview

We developed a decision-analysis model to compare the cost-effectiveness of single-dose antibiotic prophylaxis to that of a multiple-dose perioperative regimen during the surgical treatment of closed fractures. The analysis was performed from a healthcare payer perspective using 60-day and 1-year time horizons. Probabilities, costs, and health-related quality of life outcome data were obtained from the authors' institution and estimated from the literature (Table 1).

**Table 1.** Model variables

Variable	Base case	Range	References
Incidence of SSI			
Single-dose prophylaxis	0.025	0.01–0.06	Garotta et al. 1991, Gatell et al. 1987, Slobogean et al. 2008
Superficial	0.66	**^a^**	Slobogean et al. 2008
Deep	0.34	0.3–0.6	Slobogean et al. 2008
Multiple-dose prophylaxis	0.02	0.01–0.06	Garotta et al. 1991, Slobogean et al. 2008, Ali and Raza 2006
Superficial	0.57	**^a^**	Slobogean et al. 2008
Deep	0.43	0.3–0.6	Slobogean et al. 2008
Incidence of Abx-related *C. difficile*			
Risk per dose of 1st generation cephalosporin	0.00028	0.0001–0.0013	Anand et al. 1994
Doses to treat superficial SSI	40	28–56	
Risk of *C. difficile*	0.011	0.008–0.016	
Doses to treat deep SSI	126	84–168	
Risk of *C. difficile*	0.035	0.023–0.047	
Costs (2007 USD)			
ORIF ankle fracture	2,481	2,000–10,000	Bhandari et al. 2004
Preparation and dose of cefazolin	9	5–15	Garrelts et al. 1994**^b^**
Single-dose prophylaxis	9	5–15	
Multiple-dose prophylaxis	36	20–60	
Superficial SSI	2,319	500–5,000	Zoutman et al. 1998
Deep SSI	5,255	3,000–15,000	Zoutman et al. 1998
*C. difficile*	4,689	2,270–14,717	Kyne et al. 2002, O'Brien et al. 2007
Quality Adjusted Life Days (QALD)			
Ankle fracture	272	204–285	Bhandari et al. 2004
*C. difficile*	268	124–284	Kyne et al. 2002, Pepin et al. 2005
Superficial SSI	272	204–285	
Superficial SSI and *C. difficile*	268	124–284	Kyne et al. 2002, Pepin et al. 2005
Deep SSI	267	123–283	Kuntz et al. 2000
Deep SSI and *C. difficile*	263	122–282	Kyne et al. 2002, Pepin et al. 2005, Kuntz et al. 2000
**^a^** 1 – probability of deep infection.			
**^b^** cost obtained from institution's pharmacy.			
SSI: surgical site infection.			

### Model design

**Figure 1.**
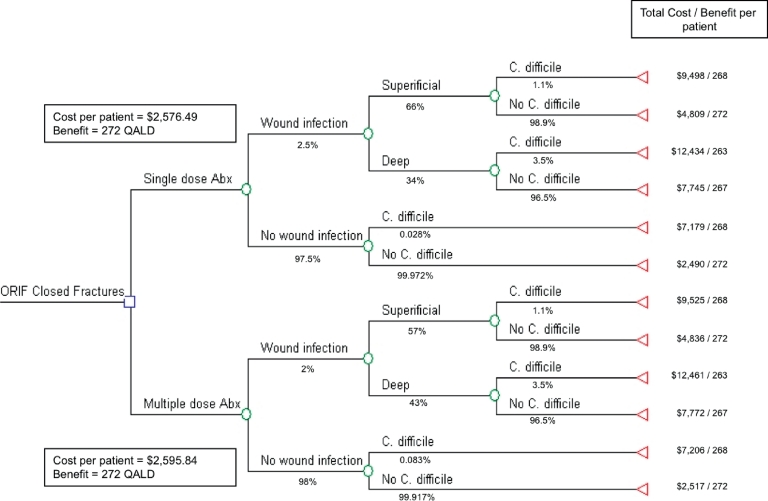
Decision tree representing the single- or multiple-dose prophylaxis decision for the surgical treatment of a closed fracture. The probability of an event occurring is listed beneath each respective branch.

A decision tree reflecting the choice of antibiotic prophylaxis and possible perioperative outcomes was created using Treeage Pro 2007 (Treeage Software Inc., Williamstown, MA) (Figure 1). We felt that surgical site infection and *Clostridium difficile*-associated diarrhea (CDAD) were potential perioperative complications that could vary based on the initial choice of prophylaxis (Anand et al. 1994). The decision tree reflects the interaction of the following outcomes: (1) wound infection and CDAD; (2) wound infection and no CDAD; (3) no wound infection but with CDAD; and (4) no wound infection and no CDAD.

Antibiotic prophylaxis is based on intravenous administration of 1 g cefazolin, with the multiple-dose regime consisting of 4 perioperative doses. Superficial surgical site infections are treated with 10 days of oral antibiotics (40 doses, cephalexin 500 mg four times daily), and deep wound infections receive surgical debridement and antibiotic coverage for 6 weeks (126 doses, cefazolin 1 g three times daily). The model assumes that wound infections and CDAD are treated without recurrence. It also assumes that the prophylaxis regimen chosen does not alter the fracture union rate in either treatment arm.

### Perioperative probabilities

#### Surgical site infection.

The probability of developing a wound infection was estimated from a recently reported meta-analysis of single-dose versus multiple-dose antibiotic prophylaxis for surgical fixation of closed fractures (Slobogean et al. 2008). The incidence of surgical site infection was estimated to be 2.5% for single-dose prophylaxis and 2.0% for multiple doses, with no statistically significant difference between the 2 prophylactic regimes (risk ratio: 1.24; 95% CI: 0.60–2.60). Pooled estimates for the distribution of superficial and deep wound infections were also used (Table 1).

#### C. difficile associated diarrhea.

The probability of developing CDAD reflects an estimated antibiotic dose-dependent risk. Anand et al. (1994) reported the incidence of C. difficile infection following the administration of cephalosporins. First-generation cephalosporins had the lowest incidence of CDAD (0.03% per dose administered). The probability of developing CDAD for each branch of the decision tree is equal to the product of the number of antibiotic doses received and the risk per dose administered (Table 1).

### Reference case

In accordance with consensus recommendations, we conducted the cost-effectiveness analysis using a reference case (Siegel et al. 1996, Weinstein et al. 1996). The reference case analysis was based on a healthy 52-year-old male undergoing operative fixation of an unstable Weber B lateral malleolus fracture of the ankle.

### Outcomes

#### Health Related Quality of Life (HRQoL).

We evaluated the effectiveness of each prophylactic intervention using utility data obtained from the literature. Utilities reflect preference-weighted measures of quality of life and are commonly generated by standard gamble or time-tradeoff techniques; generic utility measurement tools are also frequently used (Torrance 1987, Bell et al. 2001). Quality adjusted life-years (QALYs) can then be calculated from these data by summing the products of the utility for a given health state and the duration of time spent in each health state (Torrance 1987, Bell et al. 2001). Since 60-day and 1-year time horizons were used, and the time spent in each health state was measured in days, quality-adjusted life days (QALDs) are reported instead of QALYs. Within the 60-day and 1-year time horizon models, the maximum possible QALDs were 60 and 365, respectively.

The QALDs gained for each respective branch of the tree were estimated from relevant published literature (Table 2). Bhandari et al. (2004) reported utilities of 0.34 and 0.78, respectively, for the immediate and one-year postoperative periods in a cohort of operatively treated ankle fractures. The postoperative ankle fracture health state was estimated as a weighted average of these two utilities, with an individual spending 30 days in the 0.34 health state and the remainder of the time horizon in the higher 0.78 utility health state.

**Table 2.** Health-related quality of life adjustments

Event	Health state (utilities)	QALD
Ankle fracture	0.34 for 30 days 0.78 for remainder of time horizon	272
*C. difficile*	0.30 for 7 days 0.34 for 30 days 0.78 for remainder of time horizon	268
Superficial SSI	0.34 for 30 days 0.78 for remainder of time horizon	272
Superficial SSI and *C. difficile*	0.30 for 7 days 0.34 for 30 days 0.78 for remainder of time horizon	268
Deep SSI	0.30 for 10 days 0.34 for 30 days 0.78 for remainder of time horizon	267
Deep SSI and *C. difficile*	0.25 for 7 days 0.30 for 10 days 0.34 for 30 days 0.78 for remainder of time horizon	263
QALD: quality-adjusted life-days;		
SSI: surgical site infection.		

Pepin et al. (2005) recently reported that *C. difficile*-associated diarrhea resulted in a mean hospital stay of 7.0 days in a cohort of 1,125 patients aged 18–64 years with no important co-morbidities. The HRQL associated with CDAD was estimating based on the utility associated with being hospitalized (Kuntz et al. 2000).

To estimate the effect of a surgical site infection, we felt that a superficial infection would not reduce the QALDs gained. However, deep wound infection has been associated with a mean increase in length of stay of 10 days (Zoutman et al. 1998), and the associated utility for hospitalization was again used to quantify this health state (Kuntz et al. 2000).

For health states that involved multiple perioperative complications, the total QALDs gained was estimated by summing the utilities for each health state sequentially rather concurrently. For example, an individual who develops a deep wound infection and CDAD spends 10 days in the deep wound infection health state, then 7 days in the CDAD state, before completing the remainder of the model's time horizon in the weighted postoperative ankle fracture state (Table 2).

### Costs

All costs are reported in 2007 US dollars and were inflated as necessary using the Consumer Price Index inflation calculator (available online, http://www.bls.gov/cpi/). The mean costs for treatment of an ankle fracture, *C. difficile*-associated diarrhea, and superficial or deep wound infection were obtained from published reports (Anand et al. 1994, Zoutman et al. 1998, Kyne et al. 2002, O'Brien et al. 2007). The cost per dose of antibiotic prophylaxis included the costs of materials and preparation as reported by Garrelts et al. (1994), and the current pharmaceutical cost of cefazolin in our hospital (Table 1).

### Sensitivity analysis

Sensitivity analysis allows researchers to explore the impact of uncertainty on their results, and is an important component of economic analyses (Walker and Fox-Rushby 2001). One-way sensitivity analysis alters the value of a single variable over a clinically plausible range to determine its effect on the model's outcome. If the result of the analysis changes significantly when the variable is altered, then the model is “sensitive” to its value. Conversely, if sensitivity analysis does not alter the results of the cost-effectiveness analysis, then one may be confident that the results from the analysis will be stable over most clinically plausible ranges. Probabilistic sensitivity analysis using Monte Carlo simulation techniques is another method for assessing the robustness of results (Doubilet et al. 1985). In this type of analysis, the model calculations are repeated several thousand times, with differing values for each variable selected from a plausible distribution.

One-way sensitivity analysis was performed for each event probability and outcome variable based on the clinically plausible ranges described in Table 1. Probabilistic sensitivity analysis was performed using a Monte Carlo simulation of 100,000 trials. Event probabilities and outcomes were sampled from a triangular distribution using the base case and range values described in Table 1. A threshold willingness to pay (WTP) of $137 per incremental QALD ($50,000 per QALY) gained was used to choose a preferred strategy.

## Results

The quality adjusted life days (QALDs) associated with each possible health state were equivalent, and the results of the analysis suggested that differences between the two prophylactic regimens were based on cost (Table 3). These results were observed in both the 60-day and the 1-year time horizons. Open reduction and internal fixation of a closed ankle fracture using a single-dose prophylaxis strategy was found to be associated with a cost of $2,576 and 272 QALDs in a 1-year time horizon (and 34 QALDs in a 60-day time horizon). Using multiple-dose antibiotic prophylaxis resulted in a cost of $2,596 and, similarly, 272 QALDs in a 1-year time horizon (and 34 QALDs in a 60-day time horizon).

**Table 3.** Base case analysis

Prophylaxis	Cost ($)	QALD	ICER
Single	2,576.49	272	–
Multiple	2,595.84	272	Dominated
QALD: quality-adjusted life-days;			
ICER: incremental cost-effectiveness ratio.			

One-way sensitivity analysis of each variable throughout its clinically plausible range showed the results to be stable for most model variables. However, the preferred strategy was sensitive to two variables related to receiving single-dose prophylaxis: (1) the probability of surgical site infection (pSSI), and (2) the probability of a deep wound infection. Multiple-dose prophylaxis is preferred when the pSSI for the single-dose arm exceeds 3%; it is also preferred when the single-dose deep wound infection proportion is greater than 55%. These results are based on a multiple-dose pSSI of 2% and a deep wound infection proportion of 43% (Table 1).

**Figure 2.**
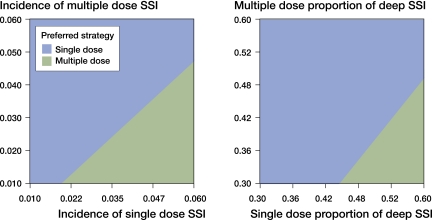
Results of two-way sensitivity analysis. The incidence of surgical site infection (A) and the proportion of deep wound infection (B) for each prophylaxis strategy is varied throughout the range described in Table 1.

Two-way sensitivity analysis was also performed by simultaneously varying the pSSI for the single- and multiple-dose strategies. Based on the willingness to pay of $137 per incremental QALD ($50,000 per QALY), one can determine the preferred strategy with different combinations of pSSI for single- and multiple-dose prophylaxis (Figure 2A). Figure 2B allows similar analysis while simultaneously varying the proportion of deep wound infection for each prophylactic strategy.

**Figure 3.**
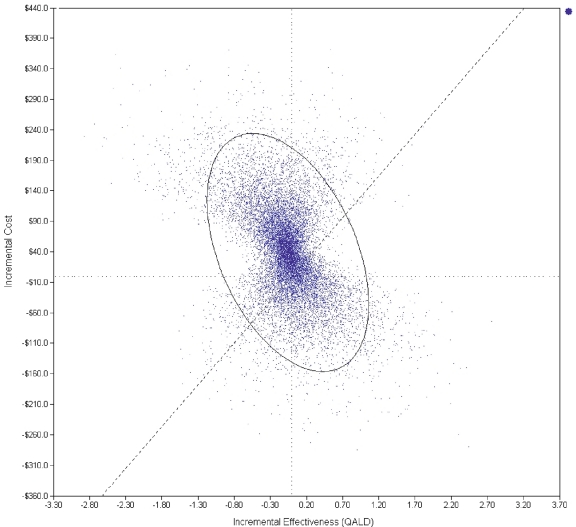
Results of Monte Carlo probabilistic sensitivity analysis. The incremental cost-effectiveness ratio for multiple-dose prophylaxis compared to the single-dose strategy is shown. A “willingness to pay” threshold of $137 per incremental QALD (dashed line) and the 95% confidence interval (ellipse) are also shown.

Probabilistic sensitivity analysis also suggested that the single-dose strategy is preferred most often. Figure 3 shows the incremental cost-effectiveness ratio (ICER) for the multiple-dose strategy compared to single-dose prophylaxis for each Monte Carlo simulation. Approximately 70% of the simulations resulted in an ICER above the $137 per QALD threshold, and most of these results were located in the “dominated” upper-left quadrant (where multiple-dose is more expensive and less effective). Consequently, increasing the willingness to pay threshold did not alter the result.

Further analysis was also performed to determine which variables most influenced the model's overall costs and effectiveness. As expected, the cost of treating an ankle fracture and the utility associated with the ankle fracture health state were found to have the greatest influence on the extent of either intervention's cost-effectiveness.

## Discussion

Our cost-effectiveness analysis was performed to help determine the optimum prophylaxis strategy during the treatment of closed long bone fractures. Our results suggest that single-dose prophylaxis is more cost-effective than a multiple-dose strategy; however, the small difference between the two strategies is primarily based on a narrow difference in cost. These results are sensitive to the incidence of wound infection in the single-dose group and its resulting proportion of deep wound infections. However, if the incidence of wound infections between the two strategies is similar, as suggested by several meta-analyses, then the results of this model remain robust.

We have not found any previous reports comparing the cost-effectiveness of two commonly used prophylactic antibiotic regimens in perioperative orthopedic care. The results of this study are consistent with previous clinical trial and meta-analysis data suggesting that the efficacies of the prophylactic strategies are similar, and complement these reports by comparing the strategies based on their cost-effectiveness. Our results also outline the clinical variables that determine when one prophylactic strategy would be preferred.

Despite the fact that we considered the additional costs associated with treating surgical wound infections or *C. difficile*-associated diarrhea, the difference in cost between the strategies was approximately $20. In addition, the quality-adjusted life-days for each prophylactic decision were estimated to be equal in both time horizon models. The similar costs and QALDs gained for each strategy occurred for several reasons: (1) wound infection is a relatively infrequent complication; (2) important adverse events related to prophylactic antibiotics (such as C. difficile-associated diarrhea) are extremely uncommon; (3) the time spent in health states associated with an adverse event (lower utility) is typically short-lived. Thus, even when using a substantially shorter time horizon, the QALDs for each prophylactic strategy remain equal.

When clinical events are uncommon, decision analysis offers the benefit of extensive sensitivity analysis. One particular strength of our report is that the results were scrutinized by performing one-way sensitivity analysis on all model variables, and further two-way analysis on selected variables. In addition, the more sophisticated Monte Carlo method of simulation allowed all variables to be varied over 100,000 repetitions. Finally, using two different time horizon models, we were able to determine that the differences between the strategies were not influenced by the time horizon considered. As a result of using multiple methods of sensitivity analysis, the stability of the results and its limitations were demonstrated. A further strength of our study was the ability to use wound infection rates from meta-analysis data (rather than a single trial) since the incidence of wound infection was a sensitive variable.

Despite the relative strength of our analysis, its results should be interpreted in the context of the model design. We based the utilities associated with each health state on relevant estimates used in previous studies; we found no published literature that directly quantified the utility associated with postoperative wound infection or antibiotic-associated *C. difficile* diarrhea. This lack of available data highlights the need for additional research to define the effect of postoperative complications on patient outcomes and healthcare costs in orthopedic trauma.

In summary, assuming similar infection rates between the prophylactic groups, this analysis suggests that single-dose prophylaxis is slightly more cost-effective than multiple-dose regimens for the treatment of closed fractures. This result is based mainly on cost, and extensive sensitivity analysis has demonstrated these results to be stable using published meta-analysis infection rates. Further work to collect utility data in the postoperative period, particularly from patients experiencing complications, would be of benefit to clinicians and health economists.
